# Protocol for a qualitative study to identify strategies to optimise hospital ePrescribing systems

**DOI:** 10.1136/bmjopen-2020-044622

**Published:** 2021-01-13

**Authors:** Catherine Heeney, Stephen Malden, Aziz Sheikh

**Affiliations:** Centre for Medical Informatics, The University of Edinburgh Usher Institute of Population Health Sciences and Informatics, Edinburgh, UK

**Keywords:** health informatics, public health, qualitative research, organisational development

## Abstract

**Introduction:**

Electronic prescribing (ePrescribing) is a key area of development and investment in the UK and across the developed world. ePrescribing is widely understood as a vehicle for tackling medication-related safety concerns, improving care quality and making more efficient use of health resources. Nevertheless, implementation of an electronic health record does not itself ensure benefits for prescribing are maximised. We examine the process of optimisation of ePrescribing systems using case studies to provide policy recommendations based on the experiences of digitally mature hospital sites.

**Methods and analysis:**

Qualitative interviews within six digitally mature sites will be carried out. The aim is to capture successful optimisation of electronic prescribing (ePrescribing) in particular health systems and hospitals. We have identified hospital sites in the UK and in three other developed countries. We used a combination of literature reviews and advice from experts at Optimising ePrescribing in Hospitals (eP Opt) Project round-table events. Sites were purposively selected based on geographical area, innovative work in ePrescribing/electronic health (eHealth) and potential transferability of practices to the UK setting. Interviews will be recorded and transcribed and transcripts coded thematically using NVivo software. Relevant policy and governance documents will be analysed, where available. Planned site visits were suspended due to the COVID-19 pandemic.

**Ethics and dissemination:**

The Usher Research Ethics Group granted approval for this study. Results will be disseminated via peer-reviewed journals in medical informatics and expert round-table events, lay member meetings and the ePrescribing Toolkit (http://www.eprescribingtoolkit.com/)—an online resource supporting National Health Service (NHS) hospitals through the ePrescribing process.

**Strengths and limitations of this study**

- A strength of this study is the way in which it contextualises the question of what works in electronic prescribing (ePrescribing) optimisation using a case study approach.
- By targeting the most advanced sites, the study could miss interesting developments in sites that may have nevertheless made significant and useful progress in improving their ePrescribing process.
- This study will involve both patient representatives and experts in clinical informatics throughout the project to guide research questions and add context to the research findings.
- It will not be possible to conduct direct observations of optimised medication prescribing processes in action, which will limit data triangulation.
- The study forms part of a wider multiphase research project that will collectively give a nuanced understanding of optimisation in ePrescribing.

## Background

International interest from private sector vendors and governments in electronic prescribing (ePrescribing) in hospitals over the last two decades can be understood as part of a wider initiative to develop eHealth capabilities in economically developed countries.^1^ The UK Department of Health and Social Care allocated £68 million for the period of 2018–2021 to further develop and improve electronic prescribing and medicines administration systems in NHS trusts. In the NHS Long Term Plan,^2^ trusts are encouraged to significantly enhance their digital capacity with regard to ePrescribing by 2029. In many UK hospitals, significant digital infrastructure exists even though it is not used to its full capacity. While in others, large-scale implementation of new electronic systems will be required.^3^ Much of the investment in ePrescribing is predicated on the hope that it will ultimately improve safety, quality and efficiency across medicines administration pathways.^4–6^ Making prescribing information legible and sharable for those involved addresses the conditions that have led to the problem of adverse drug events.^7 8^ An advantageous by-product of increased digitisation is the accompanying increase in the availability, variety and quality of data, which can be used for monitoring and evaluation purposes.^2^ Digitisation also makes data useable for a variety of potential purposes such as clinical research as well as sophisticated population health management.^2^

The Wachter Review was commissioned to look at the lessons learnt from the perceived failures of the National Programme for IT (NPfIT), which ran from 2002 for 9 years with the aim of digitising secondary care.^9^ The Wachter Review emphasised that ‘Going live with a health IT system is the beginning, not the end’.^10^ After the ‘Go Live’ and the subsequent stabilisation of the electronic health record (EHR), optimisation is the process of making adjustments to improve the system in situ. System optimisation in the context of health information technology has been described as ‘the organisational efforts to maximise the benefits and minimise the risks of utilising digital infrastructure to plan and deliver care’.^11^ Problems with the lack of integration of various types of ePrescribing systems have been highlighted as potential threats to safety.^6^ The integration of numerous information sources into clinical decision support (CDS) for ePrescribing was designed to ensure things like harmful drug–drug interactions and contraindications for particular patients with regard to specific drugs could be available at the point of prescribing.^6^ This in itself potentially creates safety challenges if clinical staff are distracted and delayed by alerts.^3^ On the one hand, an integrated system offers synchronisation between hospital activities, but in some cases, the variety of information, which can now be fed into the CDS, can worsen the problem of alert fatigue.^8^ Therefore, updates and perceived improvements to ePrescribing systems may introduce as well as mitigate risks.^3^ Despite significant investment and progress, the quality and functionality of digital health infrastructure and the integration of ePrescribing functionalities within these systems remain variable.^10^ Questions regarding integration of different hospital systems and the ability of staff to interact with changing functionalities continue to raise safety concerns.^6^

Previous work has identified a typology of optimisation targets falling within three broad areas. These are misalignment, enhancement and developing user capabilities.^12^ Misalignment is the mismatch of what an organisation needs the ePrescribing system to do and what it is capable of doing—its functionality. This can be addressed not only by configuring the system, adopting hardware and supporting infrastructure but also by changing existing ways of working. Enhancement seeks to improve the system by extending its reach in terms of integration of new information sources or ensuring more of the system’s existing functionality is employed. In developing user capabilities with the ePrescribing system, healthcare organisations typically focus on areas such as staff motivation, competence and training. Variability between different implementations has been attributed to the need for a process of convergence between technology and local organisational characteristics.^12^

A qualitative study within four early adopter sites in the USA following the implementation of an ePrescribing system (referred to as computerised provider order entry systems in the US context) aimed to identify important lessons that future implementers should consider.^13^ The findings indicated that successful implementation was accompanied by adequate user training, clear governance procedures and pre-emptive action with regard to user perceptions and the fear of change.^13^ Additionally, a questionnaire-based study identified important factors associated with successful implementation of ePrescribing systems in English hospitals, reporting similar results.^14^ Specifically, respondents indicated increased guidance around system choice, implementation strategy and standards, in addition to the support of top-level management to ensure projects were adequately resourced.^14^

Potentially, the introduction of ePrescribing can do more than simply transferring practices involved in a paper-based prescribing system into an electronic form; it can also provide opportunities for innovative ways of working.^8^ Much of what makes an ePrescribing system yield the desired functionality at local level depends on having the necessary informatics and clinical and pharmacy staff available and engaged in the complex work of configuration.^8^ However, extensive configuration of systems as a local level raises questions of the scalability of successful ePrescribing optimisation processes and practices. Optimisation, therefore, can be a process of balancing national policies, negotiations with EHR vendors and local and organisational factors.^12^ The current situation of different vendors and informatics teams configuring systems in one or more hospitals raises questions of interoperability and shared information governance across the NHS. One of the recommendations made by the Wachter Review was not to overcorrect for the perceived drawbacks of the centralised approach of the NPfIT.^10^ The goals of interoperability, shared standards and data reuse and sharing to support both personalised care and research remain central to the vision for the NHS.^2 15^

### eP OPT Project

In general, we can assume that digital maturity is an important attribute of hospitals, which can be used as benchmarks for advanced ePrescribing practices and processes. This is because their longer history of digitisation potentially provides more time post implementation to make interventions to ePrescribing systems and assess their impact—provided the requisite resources and expertise have been available. It is assumed here that dissemination of successful optimisation strategies adopted by digitally mature sites—both locally and internationally—is likely to be helpful to those currently seeking to make similar improvements to their systems. We acknowledge, however, that the difference between implementation and optimisation may not always be clear. Adequately resourced later adopters may be in a position to implement systems, such as closed-loop medication, which have been part of an optimisation process for those with a longer digital history. Indeed, in the UK Global Digital Exemplars (GDE) Programme, there have been proactive strategies employed to ensure that later adopters of electronic health systems learn lessons that have taken a number of years for their earlier adopting counterparts to learn.^16^ In this context, exemplars are digitally advanced NHS trusts who can share their learning with other trusts to improve their digital and informatics capabilities. Moreover, integrating an ePrescribing system into secondary care is a complex process likely involving different professionals and systems, as well as adjustments to fit local, specialisation-specific and policy requirements.

The Optimising ePrescribing in Hospitals (eP Opt) Project was conceived in response to the complexities involved in optimising ePrescribing systems, coupled with recent recommendations for further digitisation within NHS hospitals.^2 17–19^ The qualitative study described here is part of this larger project, the aim of which is to comprehensively investigate how best to optimise hospital ePrescribing systems through four distinct study phases.

This paper presents the study protocol for Phase 2 of the project and will detail how it contributes to a comprehensive investigation of approaches to optimise ePrescribing systems in English hospitals within the wider eP Opt Project.

## Methods and study design

A model of allowing more digitally mature hospitals to lead the way in the digitisation of healthcare has developed as a strategy in revealing useful insights to aid future health IT programmes.^8^ This model has been embodied by the GDE Programme.^11^ Our approach within eP Opt will involve examining the practices and processes of digitally mature hospitals. Their experiences will act as benchmarks for making improvements to ePrescribing systems to be adopted by sites across the NHS.

### Study aims and objectives

The aim of the qualitative study will be achieved through the following objectives:

1. Identifying and gaining access to digitally mature sites that have extensively optimised the EHR/ePrescribing systems.
2. Gain in-depth accounts of experiences with optimisation strategies from key members of staff within these sites.
3. Contrast responses within and between study sites and between UK-based and international sites.

#### Study interaction between wider project phases

**Figure 1:**
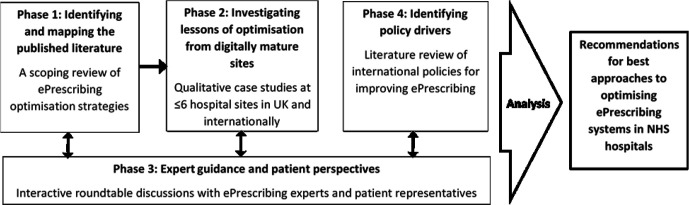
The four phases of the EP Opt Project and interactions between phases, demonstrating how Phase 2 is situated within the wider project. The project is intended to provide recommendations relevant to the UK National Health Service (NHS).

This study will be conducted and reported in accordance with the consolidated criteria for reporting qualitative research checklist.^20^ The study presented in this protocol is a standalone research project but will interact with the wider project contributing to a comprehensive understanding of the range of approaches taken to optimising ePrescribing systems in NHS hospitals. A brief description of how each project phase compliments this current study is given below and summarised in figure 1.

*Phase 1: A scoping review of optimisation strategies for ePrescribing in hospitals*. Published literature reporting on interventions to optimise ePrescribing systems in Organisation for Economic Cooperation and Development (OECD) countries was collated using a scoping review methodology.^21–23^ Results were then mapped to assess the various different optimisation strategies used and identify effective interventions. This phase assisted in the identification of leading sites to explore further in this present study (Phase 2), as well as geographical areas with higher levels of activity in optimisation of ePrescribing. A detailed description of Phase 1 is provided elsewhere.^24^

*Phase 3: Expert round-table discussions*. From our initial roundt-able events, it has emerged that the difficulties around stating exactly what the optimisation of ePrescribing entails present challenges in defining what would constitute doing this successfully. The tension between allowing local innovation to flourish with highly configurable systems and interoperability and sharing across the NHS as a whole was highlighted. Caution was urged on a number of topics including the idea that lessons from one context can easily be applied in another, wherein cultural, infrastructural and even regulatory parameters may be impactful. This applies also to the scalability of lessons in the NHS and potential financial constraints faced by some UK sites. It may not be feasible for NHS trusts to emulate some of the successful strategies found in other national contexts where different funding models are in operation. These insights have informed research questions for this present study (Phase 2) and the interview topic guide used during data collection and will shape how the results are turned into recommendations.

*Phase 4: Literature review of international ePrescribing policy*. A literature review of academic literature and grey literature around international policies relating to optimising ePrescribing capabilities across OECD countries. This phase will demonstrate local and national attempts to implement and adopt policies to optimise ePrescribing systems. Again, the end-point is to consider lessons to be applied in the UK policy context.

### Selection of case study sites and participant recruitment

In order to identify key, digitally mature sites to be approached as case studies, we will employ the following strategies. First, key publications identified in Phase 1 of the eP Opt Project will be used to identify healthcare institutions that have a major focus on developing/evaluating optimised ePrescribing systems and practices internationally. Second, Phase 3, which runs throughout the life course of the eP Opt Project, enables us to have access to experts in UK and international research and policy in ePrescribing who have pointed us towards relevant digitally mature sites. Additionally, during expert round-table discussions, attending clinicians provide information on innovative optimisation work being carried out in the UK and internationally. In Phase 2 of the project, we aim to build on previous work on ePrescribing by looking specifically at optimisation. Sites within the UK and other OECD countries have been selected on the basis of evidence of digital maturity, innovation and success in ePrescribing. These sites can be expected to have been using electronic systems for medicine management for 5–15 years, in which time they will have had ample opportunity to make improvements to the system.

Once agreements in principle had been gained from the initial site contacts, we will then employ a purposive sampling strategy to recruit staff from each site who have been involved in aspects of optimising ePrescribing systems and system users. Semistructured interviews will be carried out with a range of professionals involved in the optimisation of ePrescribing including doctors, nurses, pharmacists, IT specialists/data analysts, managers and representatives of EHR system supplier. We aim to carry out approximately 10 qualitative key informant interviews within each benchmark site (approximately 60 interviews in total). Initial contacts at each site were asked to provide email addresses for relevant staff members who are involved in system optimisation, before emails were then sent explaining the project and providing information sheets. If intent to participate was then indicated, participant consent forms were provided, and interviews were arranged.

#### Data collection

Qualitative semistructured interviews will be used to explore participants’ experiences with optimising and using ePrescribing systems within their respective sites. Interviews will be guided by an interview topic guide and will specifically seek to elicit interviewees’ experiences of improving or adjusting an existing EHR system for ePrescribing purposes, staff deployment/training, governance, specific examples of optimisation and policy drivers. We will also explore the boundary between the implementation and optimisation process, the problems that optimisation strategies have attempted to solve, experience with suppliers and unexpected consequences of making improvements to the system. Originally, face-to-face interviews were planned; however, the impact of the COVID-19 pandemic has meant that all data collection will now take place virtually using tele/videoconferencin software. Therefore, planned direct observation of ePrescribing practices at study sites will also not be possible.^25^ Interviews will be undertaken by two researchers with extensive training and experience in conducting qualitative research. All participants will be provided with information sheets and asked to sign informed consent forms prior to the interview. It will be made clear that participation in all or part of the interview is voluntary. All interviews will be audio recorded. All audio files will be transcribed verbatim by a professional transcription company.

#### Analysis

Transcripts of interviews will be analysed using inductive thematic analysis,^26^ whereby coding will be applied to data to identify emerging themes and generate new theory. After familiarising themselves with the dataset, two researchers (CH and SM) will independently code two transcripts before discussing any discrepancies in the assignment of codes, to develop and refine the coding framework and establish intercoder consistency.^27 28^ Both researchers will then use the finalised coding framework to code the remaining transcripts, before reconvening to group the emerging codes into broader themes and subthemes. The researchers will employ prospective reflexivity during data collection and analysis to reduce the impact of bias on both the data collected and the interpretation of the findings.^29^ Data analysis will be conducted using NVivo V.12 Pro qualitative data analysis software.

### Patient and public involvement

A major component of the eP Opt Project is the involvement of patient and public representatives across the four project phases. Specifically, two patient and public involvement (PPI) representatives are involved as team members, who attend research meetings and public events to provide feedback and suggestions on the work within each phase from a patient’s perspective. They have also been extensively involved with assisting with the design of an upcoming PPI round-table event for Phase 3 of the eP Opt Project, where current progress of the present study will be shared with a group of invited patients and their feedback gathered to be used as the study progresses. These round-table events and PPI consultations have helped in the formulation of research questions and will feed into analysis of the data by highlighting current gaps in practice from a patient perspective.

### Study timeline

Benchmark site recruitment started in autumn 2019. All six identified sites were initially contacted by the end of 2019, and access has been granted to begin recruitment and conduct interviews in four of the six sites. At present, we plan to continue recruitment for sites and individuals iteratively until at least 10 participants have been recruited from each site. Data collection commenced in March 2020 and is anticipated to be completed by early 2021. We have experienced delays in recruitment as access to hospital staff and availability were affected by COVID-19. We aim to have data analysis completed by February 2021, with dissemination of findings anticipated in early 2021.

## Discussion

Optimisation of ePrescribing systems can be seen as an ongoing process of identifying gaps in functionality of an already implemented system and negotiating changes, which would address these gaps, and is a complex, multifaceted process. The qualitative study described in this protocol sits within a multiphase research project that aims to comprehensively investigate approaches to take when optimising ePrescribing systems and to highlight important lessons to be learnt as ePrescribing continues to be implemented at scale within the NHS.^2^ This study will gather the insight of healthcare professionals at leading benchmark sites in both the UK and internationally. All phases of the wider eP Opt Project investigate the journey from implementation of an EHR with associated ePrescribing functionality to these systems performing as an optimal resource for professionals and patients. This addresses the important question of unintended consequences of ePrescribing systems and attempts to ensure that the safety of prescribing processes is not adversely affected as improvements are made.

## Supplementary Material

Reviewer comments

Author's manuscript

## Data Availability

Data are available upon reasonable request.

## References

[1] Catwell L, Sheikh A. Evaluating eHealth interventions: the need for continuous systemic evaluation. PLoS Med 2009;6:e1000126. 10.1371/journal.pmed.1000126 19688038 PMC2719100

[2] NHS England. The NHS long term plan, 2019.

[3] Healthcare Safety Investigation Branch. Electronic prescribing and medicines administration systems and safe discharge. 11, 2019.

[4] Bell H, Garfield S, Khosla S, et al. Mixed methods study of medication-related decision support alerts experienced during electronic prescribing for inpatients at an English Hospital. Eur J Hosp Pharm 2019;26:318–22. 10.1136/ejhpharm-2017-001483 31798854 PMC6855857

[5] OECD European Observatory on Health Systems Policies. Denmark: country health profile 2019, 2019.

[6] Garfield S, Jani Y, Jheeta S. Impact of electronic prescribing on patient safety in hospitals: implications for the UK. Evaluation 2020;15:13.

[7] Cresswell KM, Mozaffar H, Lee L, et al. Safety risks associated with the lack of integration and Interfacing of hospital health information technologies: a qualitative study of hospital electronic prescribing systems in England. BMJ Qual Saf 2017;26:530–41. 10.1136/bmjqs-2015-004925 27037303

[8] Cornford T, Dean B, Savage I. Electronic prescribing in hospitals-challenges and lessons learned, 2009.

[9] Robertson A, Bates DW, Sheikh A. The rise and fall of England’s National Programme for IT. London, England: SAGE Publications Sage UK, 2011. 10.1258/jrsm.2011.11k039 PMC3206716 22048671

[10] Wachter R. Making it work: harnessing the power of health information technology to improve care in England. London, UK: Department of Health, 2016.

[11] Cresswell KM, Bates DW, Sheikh A. Ten key considerations for the successful optimization of large-scale health information technology. J Am Med Inform Assoc 2017;24:182–7. 10.1093/jamia/ocw037 27107441 PMC7654072

[12] Wiegel V, King A, Mozaffar H, et al. A systematic analysis of the optimization of computerized physician order entry and clinical decision support systems: a qualitative study in English hospitals. Health Informatics J 2020;26:1118–32. 10.1177/1460458219868650 31566464

[13] Simon SR, Keohane CA, Amato M, et al. Lessons learned from implementation of computerized provider order entry in 5 community hospitals: a qualitative study. BMC Med Inform Decis Mak 2013;13:67. 10.1186/1472-6947-13-67 23800211 PMC3695777

[14] Cresswell K, Coleman J, Slee A, et al. Correction: investigating and learning lessons from early experiences of implementing ePrescribing systems into NHS hospitals: a questionnaire study. PLoS One 2013;8. 10.1371/annotation/1cec200a-6652-449f-91e9-8effb769de1e PMC3546047 23335961

[15] National Information Board. Personalised health and care 2020: using data and technology to transform outcomes for patients and citizens: a framework for action. HM Government London, 2014.

[16] Cresswell K, Sheikh A, Krasuska M, et al. Reconceptualising the digital maturity of health systems. Lancet Digit Health 2019;1:e200–1. 10.1016/S2589-7500(19)30083-4 33323267

[17] NHS England. Nhs digital Academy, 2018.

[18] Williams R, Cresswell K. Independent evaluation of the global digital exemplar (GDE) programme, 2020.

[19] Castle-Clarke S, Hutchings R. Achieving a digital NHS.

[20] Tong A, Sainsbury P, Craig J. Consolidated criteria for reporting qualitative research (COREQ): a 32-item checklist for interviews and focus groups. Int J Qual Health Care 2007;19:349–57. 10.1093/intqhc/mzm042 17872937

[21] Arksey H, O'Malley L. Scoping studies: towards a methodological framework. Int J Soc Res Methodol 2005;8:19–32. 10.1080/1364557032000119616

[22] Levac D, Colquhoun H, O'Brien KK. Scoping studies: advancing the methodology. Implement Sci 2010;5:69. 10.1186/1748-5908-5-69 20854677 PMC2954944

[23] Munn Z, Peters MDJ, Stern C, et al. Systematic review or scoping review? Guidance for authors when choosing between a systematic or scoping review approach. BMC Med Res Methodol 2018;18:143. 10.1186/s12874-018-0611-x 30453902 PMC6245623

[24] Williams J, Bates DW, Sheikh A. Optimising electronic prescribing in hospitals: a scoping review protocol. BMJ Health Care Inform 2020;27:e100117. 10.1136/bmjhci-2019-100117 PMC7062357 31992634

[25] Flick U. Triangulation in qualitative research. A companion to qualitative research 2004;3:178–83.

[26] Silverman D. Qualitative research. sage, 2016.

[27] Thomas J, Harden A. Methods for the thematic synthesis of qualitative research in systematic reviews. BMC Med Res Methodol 2008;8:45. 10.1186/1471-2288-8-45 18616818 PMC2478656

[28] O’Connor C, Joffe H. Intercoder reliability in qualitative research: debates and practical guidelines. International Journal of Qualitative Methods 2020;19:1609406919899220.

[29] Berger R. Now I see it, now I don’t: researcher’s position and reflexivity in qualitative research. Qualitative Research 2015;15:219–34. 10.1177/1468794112468475

