# Supplementary material for: Protocol for a qualitative study to identify strategies to optimise hospital ePrescribing systems
Source: BMJ Open. 2021 Jan 13;11(1):e044622. doi: 10.1136/bmjopen-2020-044622 (PMC7812111; doi:10.1136/bmjopen-2020-044622)
Supplement: Author's manuscript [file bmjopen-2020-044622.draft_revisions.pdf]

BMJ Open is committed to open peer review. As part of this commitment we make the peer review history of every article we publish publicly available.

When an article is published we post the peer reviewers' comments and the authors' responses online. We also post the versions of the paper that were used during peer review. These are the versions that the peer review comments apply to.

The versions of the paper that follow are the versions that were submitted during the peer review process. They are not the versions of record or the final published versions. They should not be cited or distributed as the published version of this manuscript.

BMJ Open is an open access journal and the full, final, typeset and author-corrected version of record of the manuscript is available on our site with no access controls, subscription charges or pay-per-view fees (http://bmjopen.bmj.com).

If you have any questions on BMJ Open's open peer review process please email info.bmjopen@bmj.com

BMJ Open

Protocol for a qualitative study to identify strategies to optimise hospital ePrescribing systems

Journal:	*BMJ Open*
Manuscript ID	bmjopen-2020-044622
Article Type:	Protocol
Date Submitted by the Author:	09-Sep-2020

Complete List of Authors:	Heeney, Catherine; The University of Edinburgh Usher Institute of Population Health Sciences and Informatics, Centre for Medical Informatics<br>Malden, Stephen; The University of Edinburgh Usher Institute of Population Health Sciences and Informatics, Centre for Medical Informatics<br>Sheikh, Aziz; University of Edinburgh, Division of Community Health Sciences

Keywords:	Health informatics < BIOTECHNOLOGY & BIOINFORMATICS, PUBLIC HEALTH, QUALITATIVE RESEARCH

SCHOLARONE™
Manuscripts

I, the Submitting Author has the right to grant and does grant on behalf of all authors of the Work (as defined in the below author licence), an exclusive licence and/or a non-exclusive licence for contributions from authors who are: i) UK Crown employees; ii) where BMJ has agreed a CC-BY licence shall apply, and/or iii) in accordance with the terms applicable for US Federal Government officers or employees acting as part of their official duties; on a worldwide, perpetual, irrevocable, royalty-free basis to BMJ Publishing Group Ltd ("BMJ") its licensees and where the relevant Journal is co-owned by BMJ to the co-owners of the Journal, to publish the Work in this journal and any other BMJ products and to exploit all rights, as set out in our licence.

The Submitting Author accepts and understands that any supply made under these terms is made by BMJ to the Submitting Author unless you are acting as an employee on behalf of your employer or a postgraduate student of an affiliated institution which is paying any applicable article publishing charge ("APC") for Open Access articles. Where the Submitting Author wishes to make the Work available on an Open Access basis (and intends to pay the relevant APC), the terms of reuse of such Open Access shall be governed by a Creative Commons licence – details of these licences and which Creative Commons licence will apply to this Work are set out in our licence referred to above.

Other than as permitted in any relevant BMJ Author's Self Archiving Policies, I confirm this Work has not been accepted for publication elsewhere, is not being considered for publication elsewhere and does not duplicate material already published. I confirm all authors consent to publication of this Work and authorise the granting of this licence.

For peer review only

Title:

Protocol for a qualitative study to identify strategies to optimise hospital ePrescribing systems

Authors:

Catherine Heeney1 (corresponding author), Stephen Malden1, Aziz Sheikh1

Author affiliations:

1. Centre for Medical Informatics, The Usher Institute, University of Edinburgh

Corresponding Author email and address:

Dr Catherine Heeney

Centre for Medical Informatics

The Usher Institute

The University of Edinburgh

Old Medical School

Teviot Place

Edinburgh

EH8 9AG

Email: Catherine.heeney@ed.ac.uk

Word count: 3395 (excluding abstract and reference list)

For peer review only

ABSTRACT

Introduction

Electronic prescribing (ePrescribing) is a key area of development and investment in the UK and across the developed world. ePrescribing is widely understood as a vehicle for tackling medication related safety concerns, improving care quality and making more efficient use of health resources. Nevertheless, implementation of an electronic health record (EHR) does not itself ensure benefits for prescribing are maximised. We examine the process of optimisation of ePrescribing systems using case studies to provide policy recommendations based upon the experiences of digitally mature hospital sites.

Methods and analysis

Qualitative interviews within six digitally mature sites will be carried out. The aim is to capture successful optimisation of ePrescribing in particular health systems and hospitals. We have identified hospital sites in the UK and in 3 other developed countries. We used a combination of literature reviews and advice from experts at Optimising ePrescribing (eP Opt) Project roundtable events. Sites were purposively selected based on geographical area, innovative work in ePrescribing/eHealth and potential transferability of practices to the UK setting. Interviews will be recorded and transcribed and transcripts coded thematically using Nvivo software. Relevant policy and governance documents will be analysed, where available. Planned site visits were suspended due to the COVID-19 pandemic.

Ethics and dissemination

The Usher Research Ethics Group Ethics granted approval for this study. Results will be disseminated via peer-reviewed journals in medical informatics, and expert roundtable events, lay member meetings and via the ePrescribing Toolkit (http://www.eprescribingtoolkit.com/) – an online resource supporting NHS hospitals through the ePrescribing process.

ARTICLE SUMMARY

Strengths and limitations of this study

- A strength of this study is the way in which it contextualises the question of what works in ePrescribing optimisation using a case study approach.
- By targeting the most advanced sites, the study could miss interesting developments in sites that may have nevertheless made significant and useful progress in improving their ePrescribing process.
- This study will involve both patient representatives and experts in clinical informatics throughout the project to guide research questions and add context to the research findings.
- It will not be possible to conduct direct observations of optimised medication prescribing processes in action, which will limit data triangulation.
- The study forms part of a wider multi-phase research project that will collectively give a nuanced understanding of optimisation in ePrescribing.

Key words

For peer review only

Secondary Care; Pharmacy Service, Hospital; Health Information Management

BACKGROUND

International interest from private sector vendors and governments in electronic prescribing (ePrescribing) in hospitals over the last two decades can be understood as part of a wider initiative to develop eHealth capabilities in economically-developed countries [1]. The UK Department for Health and Social Care allocated £68 million for the period of 2018 to 2021 to further develop and improve electronic prescribing and medicines administration (EPMA) systems in NHS Trusts. In the NHS Long-term Plan [2], Trusts are encouraged to significantly enhance their digital capacity with regards to ePrescribing by 2029. In many UK hospitals, significant digital infrastructure exists even though it is not used to its full capacity. While in others, large-scale implementation of new electronic systems will be required [3]. Much of the investment in ePrescribing is predicated upon the hope that it will ultimately improve safety, quality and efficiency across medicines administration pathways [4-6]. Making prescribing information legible and sharable for those involved addresses the conditions that have led to the problem of adverse drug events (ADEs) [7 8]. An advantageous by-product of increased digitisation is the accompanying increase in the availability, variety and quality of data, which can be used for monitoring and evaluation purposes [2]. Digitisation also makes data useable for a variety of potential purposes such as clinical research as well as sophisticated population health management[2].

The Wachter Review was commissioned to look at the lessons learned from the perceived failures of the National Programme for IT (NPfIT), which ran from 2002 for nine years with the aim of digitising secondary care [9]. The Wachter Review emphasised that ‘Going live with a health IT system is the beginning, not the end’ [10]. After the ‘Go Live’ and the subsequent stabilisation of the EHR, optimisation is the process of making adjustments to improve the system in situ. Systems optimisation in the context of health information technology has been described as ‘the organisational efforts to maximise the benefits and minimise the risks of utilising digital infrastructure to plan and deliver care’ [11]. Problems with the lack of integration of various types of ePrescribing systems have been highlighted as potential threats to safety [6]. The integration of numerous information sources into Clinical Decision Support (CDS) for ePrescribing were designed to ensure things like harmful drug-drug interactions and contra-indications for particular patients with regard to specific drugs could be available at the point of prescribing [6]. This in itself potentially creates safety challenges if clinical staff are distracted and delayed by alerts [3]. On the one hand, an integrated system offers synchronisation between hospital activities but in some cases, the variety of information, which can now be fed into the CDS can worsen the problem of alert fatigue [8]. Therefore, updates and perceived improvements to ePrescribing systems may introduce as well as mitigate risks [3]. Despite significant investment and progress, the quality and functionality of digital health infrastructure and the integration of ePrescribing functionalities within these systems remains variable [10]. Questions regarding integration of different hospital systems and the ability of staff to interact with changing functionalities continues to raise safety concerns [6].

Previous work has identified a typology of optimisation targets falling within three broad areas. These are: Misalignment, Enhancement and Developing user capabilities [12]. Misalignment is the mismatch of what an organisation needs the ePrescribing system to do and what it is capable of doing – its functionality. This can be addressed by both configuring the system, adapting hardware

For peer review only

and supporting infrastructure, but also by changing existing ways of working. Enhancement seeks to improve the system by extending its reach in terms of integration of new information sources or ensuring more of the system existing functionality is employed. In developing user capabilities with the ePrescribing system, healthcare organisations typically focus on areas such as staff motivation, competence, and training. Variability between different implementations has been attributed to the need for a process of convergence between technology and local organisational characteristics [12].

A qualitative study within four early adopter sites in the United States (US) following the implementation of an ePrescribing system, (referred to as computerized provider order entry CPOE) systems in the US context), aimed to identify important lessons which future implementers should consider [13]. The findings indicated that successful implementation was accompanied by adequate user training, clear governance procedures, and pre-emptive action with regards to user perceptions and the fear of change [13]. Additionally, a questionnaire-based study identified important factors associated with successful implementation of ePrescribing systems in English hospitals, reporting similar results [14]. Specifically, respondents indicated increased guidance around system choice, implementation strategy and standards, in addition to the support of top-level management to ensure projects were adequately resourced[14].

Potentially, the introduction of ePrescribing can do more than simply transferring practices involved in a paper based prescribing system into an electronic form, it can also provide opportunities for innovative ways of working [8]. Much of what makes an ePrescribing system yield the desired functionality at local level depends on having the necessary informatics, clinical and pharmacy staff available and engaged in the complex work of configuration [8]. However, extensive configuration of systems as a local level raises questions of the scalability of successful ePrescribing optimisation processes and practices. Optimisation, therefore, can be a process of balancing national policies, negotiations with EHR vendors and local and organisational factors [12]. The current situation of different vendors and informatics teams configuring systems in one or more hospitals raises questions of interoperability and shared information governance across the NHS. One of the recommendations made by the Wachter Review was not to over correct for the perceived drawbacks of the centralised approach of the NPfIT [10]. The goals of interoperability, shared standards and data reuse and sharing to support both personalised care and research remain central to the vision for the NHS [2 15].

eP OPT PROJECT

In general, we can assume that digital maturity is an important attribute of hospitals, which can be used as benchmarks for advanced ePrescribing practices and processes. This is because their longer history of digitisation, potentially provides more time post implementation to make interventions to ePrescribing systems and assess their impact – provided the requisite resources and expertise have been available. It is assumed here that dissemination of successful optimisation strategies adopted by digitally mature sites - both locally and internationally – is likely to be helpful to those currently seeking to make similar improvements to their systems. We acknowledge, however, that the difference between implementation and optimisation may not always be clear. Adequately

For peer review only

resourced later adopters may be in a position to implement systems, such as closed loop medication, which have been part of an optimisation process for those with a longer digital history. Indeed, in the UK Global Digital Exemplars (GDE) programme, there have been proactive strategies employed to ensure that later adopters of electronic health systems, learn lessons that have taken a number of years for their earlier adopting counterparts to learn[16]. In this context, exemplars are digitally advanced NHS Trusts who can share their learning with other Trusts to improve their digital and informatics capabilities. Moreover, integrating an ePrescribing system into secondary care is a complex process likely involving different professionals and systems, as well as adjustments to fit local, specialisation specific and policy requirements.

The Optimising ePrescribing in Hospitals (eP Opt) project was conceived in response to the complexities involved in optimising ePrescribing systems, coupled with recent recommendations for further digitisation within NHS hospitals [2 17-19]. The qualitative study described here is part of this larger project, the aim of which is to comprehensively investigate how best to optimise hospital ePrescribing systems through four distinct study phases.

This paper presents the study protocol for Phase 2 of the project, and will detail how it contributes to a comprehensive investigation of approaches to optimise ePrescribing systems in English hospitals within the wider eP Opt project.

Methods and study design

A model of allowing more digitally mature hospitals to lead the way in the digitisation of healthcare has developed as a strategy in revealing useful insights to aid future health IT programmes [8]. This model has been embodied by the GDE Programme [11]. Our approach within eP Opt will involve, examining the practices and processes of digitally mature hospitals. Their experiences will act as benchmarks for making improvements to ePrescribing systems to be adapted by sites across the NHS.

Study aims and objectives

The aim of the qualitative study will be achieved through the following objectives:

1. Identifying and gaining access to digitally mature sites that have extensively optimised the EHR/ePrescribing systems
2. Gain in-depth accounts of experiences with optimisation strategies from key members of staff within these sites
3. Contrast responses within and between study sites, and between UK based and international sites.

Study interaction between wider project phases

This study will be conducted and reported in accordance with the Consolidated criteria for reporting qualitative research (CoreQ) checklist [20]. The study presented in this protocol is a standalone research project, but will interact with the wider project contributing to a comprehensive understanding of the range of approaches taken to optimising ePrescribing systems in NHS hospitals. A brief description of how each project phase compliments this current study is given below, and summarised in Figure 1.

For peer review only

Phase 1: A scoping review of optimisation strategies for ePrescribing in hospitals. Published literature reporting on interventions to optimise ePrescribing systems in OECD countries was collated using a scoping review methodology [21-23]. Results were then mapped to assess the various different optimisation strategies used and identify effective interventions. This phase assisted in the identification of leading sites to explore further in this present study (Phase 2), as well as geographical areas with higher levels of activity in optimisation of ePrescribing. A detailed description of Phase 1 is provided elsewhere [24].

Phase 3: Expert roundtable discussions. From our initial roundtable events, it has emerged that the difficulties around stating exactly what the optimisation of ePrescribing entails presents challenges in defining what would constitute doing this successfully. The tension between allowing local innovation to flourish with highly configurable systems and interoperability and sharing across the NHS as a whole was highlighted. Caution was urged on a number of topics including the idea that lessons from one context can easily be applied in another, wherein cultural, infrastructural and even regulatory parameters may be impactful. This applies also to the scalability of lessons in the NHS and potential financial constraints faced by some UK sites. It may not be feasible for NHS trusts to emulate some of the successful strategies found in other national contexts where different funding models are in operation. These insights have informed research questions for this present study (Phase 2) and the interview topic guide used during data collection and will shape how the results are turned into recommendations.

Phase 4: Literature review of international ePrescribing policy. A literature review of academic literature and grey literature around international policies relating to optimising ePrescribing capabilities across OECD countries. This phase will demonstrate local and national attempts to implement and adapt policies to optimise ePrescribing systems. Again, the end-point is to consider lessons to be applied in the UK policy context.

Figure 1.

Selection of case study sites and participant recruitment

In order to identify key, digitally mature sites to be approached as case studies we will employ the following strategies. Firstly, key publications identified in Phase 1 of the eP Opt project will be used to identify healthcare institutions that have a major focus on developing/evaluating optimised ePrescribing systems and practices internationally. Secondly, Phase 3, which runs throughout the life course of the eP Opt project, enables us to access to experts in UK and international research and policy in ePrescribing who have pointed us towards relevant digitally mature sites. Additionally, during expert roundtable discussions, attending clinicians provide information on innovative optimisation work being carried out in the UK and internationally. In Phase 2 of the project, we aim to build on previous work on ePrescribing by looking specifically at optimisation. Sites within the UK and other OECD countries have been selected on the basis of evidence of digital maturity, innovation and success in ePrescribing. These sites can be expected to have been using electronic systems for medicines management for 5-15 years, in which time they will have had ample opportunity to make improvements to the system.

For peer review only

Once agreements in principle had been gained from the initial site contacts, we will then employ a purposive sampling strategy to recruit staff from each site who have been involved in aspects of optimising ePrescribing systems and system users. Semi-structured interviews will be carried out with a range of professionals involved in the optimisation of ePrescribing including: doctors, nurses, pharmacists, IT specialists/data analysts, managers and representatives EHR system supplier. We aim to carry out approximately 10 qualitative key informant interviews within each benchmark site (approximately 60 interviews in total). Initial contacts at each site were asked to provide email addresses for relevant staff members who are involved in systems optimisation, before emails were then sent explaining the project and providing information sheets. If intent to participate was then indicated, participant consent forms were provided and interviews were arranged.

Data collection

Qualitative semi-structured interviews will be used to explore participants' experiences with optimising and using ePrescribing systems within their respective sites. Interviews will be guided by an interview topic guide and will specifically seek to elicit interviewees' experiences of improving or adjusting an existing EHR system for ePrescribing purposes, staff deployment/training, governance, specific examples of optimisation and policy drivers. We will also explore the boundary between the implementation and optimisation process, the problems that optimisation strategies have attempted to solve, experience with suppliers and unexpected consequences of making improvements to the system. Originally, face-to-face interviews were planned; however, the impact of the COVID-19 pandemic has meant that all data collection will now take place virtually using tele-/video-conferencing software. Therefore, planned direct observation of ePrescribing practices at study sites will also not be possible [25]. Interviews will be undertaken by two researchers with extensive training and experience in conducting qualitative research. All participants will be provided with information sheets and asked to sign informed consent forms prior to the interview. It will be made clear that participation in all or part of the interview is voluntary. All interviews will be audio recorded. All audio files will transcribed verbatim by a professional transcription company.

Analysis

Transcripts of interviews will be analysed using inductive thematic analysis[26], whereby coding will be applied to data to identify emerging themes and generate new theory. After familiarising themselves with the dataset, two researchers (CH and SM) will independently code two transcripts before discussing any discrepancies in the assignment of codes, to develop and refine the coding framework and establish inter coder consistency[27 28]. Both researchers will then use the finalised coding framework to code the remaining transcripts, before reconvening to group the emerging codes into broader themes and sub-themes. The researchers will employ prospective reflexivity during data collection and analysis to reduce the impact of bias on both the data collected, and the interpretation of the findings [29]. Data analysis will be conducted using NVivo 12 Pro qualitative data analysis software.

Patient and public involvement

A major component of the eP Opt project is the involvement of patient and public representatives across the four project phases. Specifically, two patient and public (PPI) representatives are involved as team members, who attend research meetings and public events to provide feedback and suggestions on the work within each phase from a patient's perspective. They have also been extensively involved with assisting with the design of an upcoming PPI roundtable event for phase 3 of the eP Opt project, where current progress of the present study will be shared with a group of

For peer review only

invited patients, and their feedback gathered to be used as the study progresses. These roundtable events and PPI consultations have helped in the formulation of research questions and will feed into analysis of the data by highlighting current gaps in practice from a patient perspective.

Study timeline

Benchmark site recruitment started in autumn 2019. All six identified sites were initially contacted in by the end of 2019 and access has been granted to begin recruitment and conduct interviews in four of the six sites. At present, we plan to continue recruitment for sites and individuals iteratively until at least 10 participants have been recruited from each site. Data collection commenced in March 2020, and is anticipated to be completed by early 2021. We have experienced delays in recruitment as access to hospital staff and availability was affected by COVID-19. We aim to have data analysis completed by February 2021, with dissemination of findings anticipated in early 2021.

Ethical considerations

This study was granted ethical approval by the Usher Research Ethics Group (University of Edinburgh) on 21/01/2020 (ref.1906) and the relevant NHS research and development approvals were acquired for UK-based sites on 23/01/2020 (ref.19/HRA/7015). As this research is exclusively being conducted with healthcare professionals, there is no involvement of vulnerable groups, and therefore there is minimal ethical concern. Despite this, there are a number of data protection and privacy considerations that should be acknowledged. Firstly, due to the ongoing COVID-19 pandemic, this research project is being conducted remotely using teleconferencing software. This presents a number of potential data protection issues, such as the potential for unauthorised access to scheduled virtual meetings. This issue is being mitigated by using only University-approved accounts to set up interviews, using meeting passwords, and ensuring interviews are undertaken with both the interviewee and interviewer in a private room. Another ethical consideration is the need to protect anonymity of research participants. As we are focusing this research on well-known, world-leading institutions in health informatics, extra care should be taken when presenting research findings to not compromise the anonymity of the respondents. Therefore, specific job titles will not be used in conjunction with individual quotes, and any quotes which could be used to potentially identify someone will not be used. All audio files, transcripts and consent forms will be kept in a secure, password-protected folder within the University of Edinburgh's Datastore. Consent forms will be kept in separate folders from the rest of the research data.

Discussion

Optimisation of ePrescribing systems can be seen as an ongoing process of identifying gaps in functionality of an already implemented system and negotiating changes, which would address these gaps, and is a complex, multifaceted process. The qualitative study described in this protocol, sits within a multi-phase research project that aims to comprehensively investigate approaches to take when optimising ePrescribing systems, and to highlight important lessons to be learned as ePrescribing continues to be implemented at scale within the NHS [2]. This study will gather the insight of healthcare professionals at leading benchmark sites both in the UK and internationally. All phases of the wider eP Opt project investigate the journey from implementation of an EHR with associated ePrescribing functionality to these systems performing as an optimal resource for professionals and

For peer review only

patients. This addresses the important question of unintended consequences of ePrescribing systems, and attempts to ensure that the safety of prescribing processes is not adversely affected as improvements are made.

Author's contributions

AS conceptualised the project and designed the study. CH recruited study sites; CH and SM recruited individual participants within study sites and conducted data collection. CH prepared the first draft of the manuscript. SM and CH prepared the second draft of the manuscript. All authors contributed to the final draft of the manuscript and approved it for submission.

Acknowledgements

The Authors thank all study sites and participants who are currently participating in the project, in addition to the PPI representatives Jillian Beggs and Antony Chuter. We also thank colleagues at the Usher Institute who have contributed to the wider phases of the eP Opt project (Serena Tricarico, Toni Wigglesworth Jac Williams, Mike Holder, Uditha Perera and Matt Bouamrane).

Funding statement

This study/project is funded by the National Institute for Health Research (NIHR) [Optimising ePrescribing in Hospitals (PR-ST-01-10001)/Policy Research Programme]. The views expressed are those of the author(s) and not necessarily those of the NIHR or the Department of Health and Social Care.

Data statement

All transcriptions will be anonymised as far as is possible and transferred to the University of Edinburgh's secure Datastore at the earliest suitable time for secondary use subject to ethics permissions.

Competing interests

The authors declare no conflicts of interest

Figure 1. The four phases of the eP Opt project and interactions between phases, demonstrating how Phase 2 is situated within the wider project.

References

1. Catwell L, Sheikh A. Evaluating eHealth interventions: the need for continuous systemic evaluation. *PLoS Med* 2009;6(8):e1000126.
2. NHS England. The NHS long term plan, 2019.
3. Healthcare Safety Investigation Branch. Electronic Prescribing and Medicines Administration Systems and Safe Discharge, 2019:11.
4. Bell H, Garfield S, Khosla S, et al. Mixed methods study of medication-related decision support alerts experienced during electronic prescribing for inpatients at an English hospital. *European Journal of Hospital Pharmacy* 2019;26(6):318-22.
5. OECD European Observatory on Health Systems Policies. Denmark: Country Health Profile 20192019.

For peer review only

6. Garfield S, Jani Y, Jheeta S, et al. Impact of electronic prescribing on patient safety in hospitals: implications for the UK. *Evaluation* 2020;15:13.
7. Cresswell KM, Mozaffar H, Lee L, et al. Safety risks associated with the lack of integration and interfacing of hospital health information technologies: a qualitative study of hospital electronic prescribing systems in England. *BMJ Quality & Safety* 2017;26(7):530-41.
8. Cornford T, Dean B, Savage I, et al. Electronic prescribing in hospitals-challenges and lessons learned. 2009
9. Robertson A, Bates DW, Sheikh A. The rise and fall of England's National Programme for IT: SAGE Publications Sage UK: London, England, 2011.
10. Wachter R. Making IT work: harnessing the power of health information technology to improve care in England. *London, UK: Department of Health* 2016
11. Cresswell KM, Bates DW, Sheikh A. Ten key considerations for the successful optimization of large-scale health information technology. *Journal of the American Medical Informatics Association* 2017;24(1):182-87.
12. Wiegel V, King A, Mozaffar H, et al. A systematic analysis of the optimization of computerized physician order entry and clinical decision support systems: A qualitative study in English hospitals. *Health Informatics Journal* 2019:1460458219868650.
13. Simon SR, Keohane CA, Amato M, et al. Lessons learned from implementation of computerized provider order entry in 5 community hospitals: a qualitative study. *BMC medical informatics and decision making* 2013;13(1):67.
14. Cresswell K, Coleman J, Slee A, et al. Investigating and learning lessons from early experiences of implementing ePrescribing systems into NHS hospitals: a questionnaire study. *PLoS One* 2013;8(1)
15. National Information Board. Personalised health and care 2020: using data and technology to transform outcomes for patients and citizens: a framework for action: HM Government London, 2014.
16. Cresswell K, Sheikh A, Krasuska M, et al. Reconceptualising the digital maturity of health systems. *The Lancet Digital Health* 2019;1(5):e200-e01.
17. NHS England. NHS digital academy., 2018.
18. Williams R, Cresswell K. Independent Evaluation of the Global Digital Exemplar (GDE) Programme. 2020
19. Castle-Clarke S, Hutchings R. Achieving a digital NHS.
20. Tong A, Sainsbury P, Craig J. Consolidated criteria for reporting qualitative research (COREQ): a 32-item checklist for interviews and focus groups. *International journal for quality in health care* 2007;19(6):349-57.
21. Arksey H, O'Malley L. Scoping studies: towards a methodological framework. *International journal of social research methodology* 2005;8(1):19-32.
22. Levac D, Colquhoun H, O'Brien KK. Scoping studies: advancing the methodology. *Implementation science* 2010;5(1):69.
23. Munn Z, Peters MD, Stern C, et al. Systematic review or scoping review? Guidance for authors when choosing between a systematic or scoping review approach. *BMC medical research methodology* 2018;18(1):143.
24. Williams J, Bates DW, Sheikh A. Optimising electronic prescribing in hospitals: a scoping review protocol. *BMJ Health & Care Informatics* 2020;27(1)
25. Flick U. Triangulation in qualitative research. *A companion to qualitative research* 2004;3:178-83.
26. Silverman D. Qualitative research: sage 2016.
27. Thomas J, Harden A. Methods for the thematic synthesis of qualitative research in systematic reviews. *BMC Medical Research Methodology* 2008;8(1):45. doi: 10.1186/1471-2288-8-45
28. O'Connor C, Joffe H. Intercoder Reliability in Qualitative Research: Debates and Practical Guidelines. *International Journal of Qualitative Methods* 2020;19:1609406919899220. doi: 10.1177/1609406919899220

For peer review only

29. Berger R. Now I see it, now I don't: Researcher's position and reflexivity in qualitative research. *Qualitative research* 2015;15(2):219-34.

For peer review only

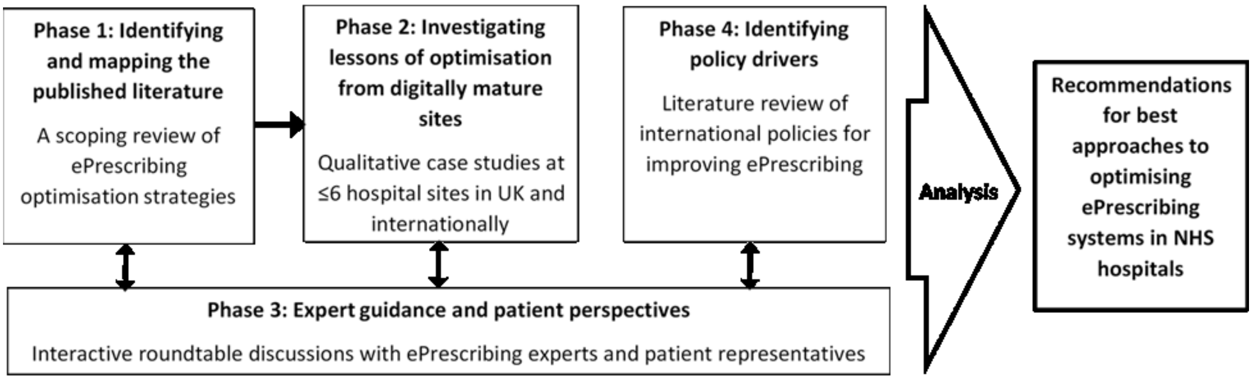

For peer review only

BMJ Open

Protocol for a qualitative study to identify strategies to optimise hospital ePrescribing systems

Journal:	*BMJ Open*
Manuscript ID	bmjopen-2020-044622.R1
Article Type:	Protocol
Date Submitted by the Author:	25-Nov-2020
Complete List of Authors:	Heeney, Catherine; The University of Edinburgh Usher Institute of Population Health Sciences and Informatics, Centre for Medical Informatics<br>Malden, Stephen; The University of Edinburgh Usher Institute of Population Health Sciences and Informatics, Centre for Medical Informatics<br>Sheikh, Aziz; University of Edinburgh, Division of Community Health Sciences
**Primary Subject Heading**:	Health informatics
Secondary Subject Heading:	Qualitative research
Keywords:	Health informatics < BIOTECHNOLOGY & BIOINFORMATICS, PUBLIC HEALTH, QUALITATIVE RESEARCH, Organisational development < HEALTH SERVICES ADMINISTRATION & MANAGEMENT

SCHOLARONE™
Manuscripts

I, the Submitting Author has the right to grant and does grant on behalf of all authors of the Work (as defined in the below author licence), an exclusive licence and/or a non-exclusive licence for contributions from authors who are: i) UK Crown employees; ii) where BMJ has agreed a CC-BY licence shall apply, and/or iii) in accordance with the terms applicable for US Federal Government officers or employees acting as part of their official duties; on a worldwide, perpetual, irrevocable, royalty-free basis to BMJ Publishing Group Ltd ("BMJ") its licensees and where the relevant Journal is co-owned by BMJ to the co-owners of the Journal, to publish the Work in this journal and any other BMJ products and to exploit all rights, as set out in our licence.

The Submitting Author accepts and understands that any supply made under these terms is made by BMJ to the Submitting Author unless you are acting as an employee on behalf of your employer or a postgraduate student of an affiliated institution which is paying any applicable article publishing charge ("APC") for Open Access articles. Where the Submitting Author wishes to make the Work available on an Open Access basis (and intends to pay the relevant APC), the terms of reuse of such Open Access shall be governed by a Creative Commons licence – details of these licences and which Creative Commons licence will apply to this Work are set out in our licence referred to above.

Other than as permitted in any relevant BMJ Author's Self Archiving Policies, I confirm this Work has not been accepted for publication elsewhere, is not being considered for publication elsewhere and does not duplicate material already published. I confirm all authors consent to publication of this Work and authorise the granting of this licence.

For peer review only

Title:

Protocol for a qualitative study to identify strategies to optimise hospital ePrescribing systems

Authors:

Catherine Heeney1 (corresponding author), Stephen Malden1, Aziz Sheikh1

Author affiliations:

1. Centre for Medical Informatics, The Usher Institute, University of Edinburgh

Corresponding Author email and address:

Dr Catherine Heeney

Centre for Medical Informatics

The Usher Institute

The University of Edinburgh

Old Medical School

Teviot Place

Edinburgh

EH8 9AG

Email: Catherine.heeney@ed.ac.uk

Word count: 3420 (excluding abstract and reference list)

For peer review only

ABSTRACT

Introduction

Electronic prescribing (ePrescribing) is a key area of development and investment in the UK and across the developed world. ePrescribing is widely understood as a vehicle for tackling medication related safety concerns, improving care quality and making more efficient use of health resources. Nevertheless, implementation of an electronic health record (EHR) does not itself ensure benefits for prescribing are maximised. We examine the process of optimisation of ePrescribing systems using case studies to provide policy recommendations based upon the experiences of digitally mature hospital sites.

Methods and analysis

Qualitative interviews within six digitally mature sites will be carried out. The aim is to capture successful optimisation of ePrescribing in particular health systems and hospitals. We have identified hospital sites in the UK and in 3 other developed countries. We used a combination of literature reviews and advice from experts at Optimising ePrescribing (eP Opt) Project roundtable events. Sites were purposively selected based on geographical area, innovative work in ePrescribing/eHealth and potential transferability of practices to the UK setting. Interviews will be recorded and transcribed and transcripts coded thematically using Nvivo software. Relevant policy and governance documents will be analysed, where available. Planned site visits were suspended due to the COVID-19 pandemic.

Ethics and dissemination

The Usher Research Ethics Group Ethics granted approval for this study. Results will be disseminated via peer-reviewed journals in medical informatics, and expert roundtable events, lay member meetings and via the ePrescribing Toolkit (http://www.eprescribingtoolkit.com/) – an online resource supporting NHS hospitals through the ePrescribing process.

ARTICLE SUMMARY

Strengths and limitations of this study

- A strength of this study is the way in which it contextualises the question of what works in ePrescribing optimisation using a case study approach.
- By targeting the most advanced sites, the study could miss interesting developments in sites that may have nevertheless made significant and useful progress in improving their ePrescribing process.
- This study will involve both patient representatives and experts in clinical informatics throughout the project to guide research questions and add context to the research findings.
- It will not be possible to conduct direct observations of optimised medication prescribing processes in action, which will limit data triangulation.
- The study forms part of a wider multi-phase research project that will collectively give a nuanced understanding of optimisation in ePrescribing.

Key words

For peer review only

Secondary Care; Pharmacy Service, Hospital; Health Information Management

BACKGROUND

International interest from private sector vendors and governments in electronic prescribing (ePrescribing) in hospitals over the last two decades can be understood as part of a wider initiative to develop eHealth capabilities in economically-developed countries [1]. The UK Department for Health and Social Care allocated £68 million for the period of 2018 to 2021 to further develop and improve electronic prescribing and medicines administration (EPMA) systems in NHS Trusts. In the NHS Long-term Plan [2], Trusts are encouraged to significantly enhance their digital capacity with regards to ePrescribing by 2029. In many UK hospitals, significant digital infrastructure exists even though it is not used to its full capacity. While in others, large-scale implementation of new electronic systems will be required [3]. Much of the investment in ePrescribing is predicated upon the hope that it will ultimately improve safety, quality and efficiency across medicines administration pathways [4-6]. Making prescribing information legible and sharable for those involved addresses the conditions that have led to the problem of adverse drug events (ADEs) [7 8]. An advantageous by-product of increased digitisation is the accompanying increase in the availability, variety and quality of data, which can be used for monitoring and evaluation purposes [2]. Digitisation also makes data useable for a variety of potential purposes such as clinical research as well as sophisticated population health management[2].

The Wachter Review was commissioned to look at the lessons learned from the perceived failures of the National Programme for IT (NPfIT), which ran from 2002 for nine years with the aim of digitising secondary care [9]. The Wachter Review emphasised that 'Going live with a health IT system is the beginning, not the end' [10]. After the 'Go Live' and the subsequent stabilisation of the EHR, optimisation is the process of making adjustments to improve the system in situ. Systems optimisation in the context of health information technology has been described as 'the organisational efforts to maximise the benefits and minimise the risks of utilising digital infrastructure to plan and deliver care' [11]. Problems with the lack of integration of various types of ePrescribing systems have been highlighted as potential threats to safety [6]. The integration of numerous information sources into Clinical Decision Support (CDS) for ePrescribing were designed to ensure things like harmful drug-drug interactions and contra-indications for particular patients with regard to specific drugs could be available at the point of prescribing [6]. This in itself potentially creates safety challenges if clinical staff are distracted and delayed by alerts [3]. On the one hand, an integrated system offers synchronisation between hospital activities but in some cases, the variety of information, which can now be fed into the CDS can worsen the problem of alert fatigue [8]. Therefore, updates and perceived improvements to ePrescribing systems may introduce as well as mitigate risks [3]. Despite significant investment and progress, the quality and functionality of digital health infrastructure and the integration of ePrescribing functionalities within these systems remains variable [10]. Questions regarding integration of different hospital systems and the ability of staff to interact with changing functionalities continues to raise safety concerns [6].

Previous work has identified a typology of optimisation targets falling within three broad areas. These are: Misalignment, Enhancement and Developing user capabilities [12]. Misalignment is the mismatch of what an organisation needs the ePrescribing system to do and what it is capable of doing – its functionality. This can be addressed by both configuring the system, adapting hardware

For peer review only - http://bmjopen.bmj.com/site/about/guidelines.xhtml

and supporting infrastructure, but also by changing existing ways of working. Enhancement seeks to improve the system by extending its reach in terms of integration of new information sources or ensuring more of the system existing functionality is employed. In developing user capabilities with the ePrescribing system, healthcare organisations typically focus on areas such as staff motivation, competence, and training. Variability between different implementations has been attributed to the need for a process of convergence between technology and local organisational characteristics [12].

A qualitative study within four early adopter sites in the United States (US) following the implementation of an ePrescribing system, (referred to as computerized provider order entry CPOE) systems in the US context), aimed to identify important lessons which future implementers should consider [13]. The findings indicated that successful implementation was accompanied by adequate user training, clear governance procedures, and pre-emptive action with regards to user perceptions and the fear of change [13]. Additionally, a questionnaire-based study identified important factors associated with successful implementation of ePrescribing systems in English hospitals, reporting similar results [14]. Specifically, respondents indicated increased guidance around system choice, implementation strategy and standards, in addition to the support of top-level management to ensure projects were adequately resourced[14].

Potentially, the introduction of ePrescribing can do more than simply transferring practices involved in a paper based prescribing system into an electronic form, it can also provide opportunities for innovative ways of working [8]. Much of what makes an ePrescribing system yield the desired functionality at local level depends on having the necessary informatics, clinical and pharmacy staff available and engaged in the complex work of configuration [8]. However, extensive configuration of systems as a local level raises questions of the scalability of successful ePrescribing optimisation processes and practices. Optimisation, therefore, can be a process of balancing national policies, negotiations with EHR vendors and local and organisational factors [12]. The current situation of different vendors and informatics teams configuring systems in one or more hospitals raises questions of interoperability and shared information governance across the NHS. One of the recommendations made by the Wachter Review was not to over correct for the perceived drawbacks of the centralised approach of the NPfIT [10]. The goals of interoperability, shared standards and data reuse and sharing to support both personalised care and research remain central to the vision for the NHS [2 15].

eP OPT PROJECT

In general, we can assume that digital maturity is an important attribute of hospitals, which can be used as benchmarks for advanced ePrescribing practices and processes. This is because their longer history of digitisation, potentially provides more time post implementation to make interventions to ePrescribing systems and assess their impact – provided the requisite resources and expertise have been available. It is assumed here that dissemination of successful optimisation strategies adopted by digitally mature sites - both locally and internationally – is likely to be helpful to those currently seeking to make similar improvements to their systems. We acknowledge, however, that the difference between implementation and optimisation may not always be clear. Adequately

resourced later adopters may be in a position to implement systems, such as closed loop medication, which have been part of an optimisation process for those with a longer digital history. Indeed, in the UK Global Digital Exemplars (GDE) programme, there have been proactive strategies employed to ensure that later adopters of electronic health systems, learn lessons that have taken a number of years for their earlier adopting counterparts to learn[16]. In this context, exemplars are digitally advanced NHS Trusts who can share their learning with other Trusts to improve their digital and informatics capabilities. Moreover, integrating an ePrescribing system into secondary care is a complex process likely involving different professionals and systems, as well as adjustments to fit local, specialisation specific and policy requirements.

The Optimising ePrescribing in Hospitals (eP Opt) project was conceived in response to the complexities involved in optimising ePrescribing systems, coupled with recent recommendations for further digitisation within NHS hospitals [2 17-19]. The qualitative study described here is part of this larger project, the aim of which is to comprehensively investigate how best to optimise hospital ePrescribing systems through four distinct study phases.

This paper presents the study protocol for Phase 2 of the project, and will detail how it contributes to a comprehensive investigation of approaches to optimise ePrescribing systems in English hospitals within the wider eP Opt project.

Methods and study design

A model of allowing more digitally mature hospitals to lead the way in the digitisation of healthcare has developed as a strategy in revealing useful insights to aid future health IT programmes [8]. This model has been embodied by the GDE Programme [11]. Our approach within eP Opt will involve, examining the practices and processes of digitally mature hospitals. Their experiences will act as benchmarks for making improvements to ePrescribing systems to be adapted by sites across the NHS.

Study aims and objectives

The aim of the qualitative study will be achieved through the following objectives:

1. Identifying and gaining access to digitally mature sites that have extensively optimised the EHR/ePrescribing systems
2. Gain in-depth accounts of experiences with optimisation strategies from key members of staff within these sites
3. Contrast responses within and between study sites, and between UK based and international sites.

Study interaction between wider project phases

This study will be conducted and reported in accordance with the Consolidated criteria for reporting qualitative research (CoreQ) checklist [20]. The study presented in this protocol is a standalone research project, but will interact with the wider project contributing to a comprehensive understanding of the range of approaches taken to optimising ePrescribing systems in NHS hospitals. A brief description of how each project phase compliments this current study is given below, and summarised in Figure 1.

For peer review only

Phase 1: A scoping review of optimisation strategies for ePrescribing in hospitals. Published literature reporting on interventions to optimise ePrescribing systems in OECD countries was collated using a scoping review methodology [21-23]. Results were then mapped to assess the various different optimisation strategies used and identify effective interventions. This phase assisted in the identification of leading sites to explore further in this present study (Phase 2), as well as geographical areas with higher levels of activity in optimisation of ePrescribing. A detailed description of Phase 1 is provided elsewhere [24].

Phase 3: Expert roundtable discussions. From our initial roundtable events, it has emerged that the difficulties around stating exactly what the optimisation of ePrescribing entails presents challenges in defining what would constitute doing this successfully. The tension between allowing local innovation to flourish with highly configurable systems and interoperability and sharing across the NHS as a whole was highlighted. Caution was urged on a number of topics including the idea that lessons from one context can easily be applied in another, wherein cultural, infrastructural and even regulatory parameters may be impactful. This applies also to the scalability of lessons in the NHS and potential financial constraints faced by some UK sites. It may not be feasible for NHS trusts to emulate some of the successful strategies found in other national contexts where different funding models are in operation. These insights have informed research questions for this present study (Phase 2) and the interview topic guide used during data collection and will shape how the results are turned into recommendations.

Phase 4: Literature review of international ePrescribing policy. A literature review of academic literature and grey literature around international policies relating to optimising ePrescribing capabilities across OECD countries. This phase will demonstrate local and national attempts to implement and adapt policies to optimise ePrescribing systems. Again, the end-point is to consider lessons to be applied in the UK policy context.

Figure 1.

Selection of case study sites and participant recruitment

In order to identify key, digitally mature sites to be approached as case studies we will employ the following strategies. Firstly, key publications identified in Phase 1 of the eP Opt project will be used to identify healthcare institutions that have a major focus on developing/evaluating optimised ePrescribing systems and practices internationally. Secondly, Phase 3, which runs throughout the life course of the eP Opt project, enables us to access to experts in UK and international research and policy in ePrescribing who have pointed us towards relevant digitally mature sites. Additionally, during expert roundtable discussions, attending clinicians provide information on innovative optimisation work being carried out in the UK and internationally. In Phase 2 of the project, we aim to build on previous work on ePrescribing by looking specifically at optimisation. Sites within the UK and other OECD countries have been selected on the basis of evidence of digital maturity, innovation and success in ePrescribing. These sites can be expected to have been using electronic systems for medicines management for 5-15 years, in which time they will have had ample opportunity to make improvements to the system.

For peer review only

Once agreements in principle had been gained from the initial site contacts, we will then employ a purposive sampling strategy to recruit staff from each site who have been involved in aspects of optimising ePrescribing systems and system users. Semi-structured interviews will be carried out with a range of professionals involved in the optimisation of ePrescribing including: doctors, nurses, pharmacists, IT specialists/data analysts, managers and representatives EHR system supplier. We aim to carry out approximately 10 qualitative key informant interviews within each benchmark site (approximately 60 interviews in total). Initial contacts at each site were asked to provide email addresses for relevant staff members who are involved in systems optimisation, before emails were then sent explaining the project and providing information sheets. If intent to participate was then indicated, participant consent forms were provided and interviews were arranged.

Data collection

Qualitative semi-structured interviews will be used to explore participants' experiences with optimising and using ePrescribing systems within their respective sites. Interviews will be guided by an interview topic guide and will specifically seek to elicit interviewees' experiences of improving or adjusting an existing EHR system for ePrescribing purposes, staff deployment/training, governance, specific examples of optimisation and policy drivers. We will also explore the boundary between the implementation and optimisation process, the problems that optimisation strategies have attempted to solve, experience with suppliers and unexpected consequences of making improvements to the system. Originally, face-to-face interviews were planned; however, the impact of the COVID-19 pandemic has meant that all data collection will now take place virtually using tele-/video-conferencing software. Therefore, planned direct observation of ePrescribing practices at study sites will also not be possible [25]. Interviews will be undertaken by two researchers with extensive training and experience in conducting qualitative research. All participants will be provided with information sheets and asked to sign informed consent forms prior to the interview. It will be made clear that participation in all or part of the interview is voluntary. All interviews will be audio recorded. All audio files will transcribed verbatim by a professional transcription company.

Analysis

Transcripts of interviews will be analysed using inductive thematic analysis[26], whereby coding will be applied to data to identify emerging themes and generate new theory. After familiarising themselves with the dataset, two researchers (CH and SM) will independently code two transcripts before discussing any discrepancies in the assignment of codes, to develop and refine the coding framework and establish inter coder consistency[27 28]. Both researchers will then use the finalised coding framework to code the remaining transcripts, before reconvening to group the emerging codes into broader themes and sub-themes. The researchers will employ prospective reflexivity during data collection and analysis to reduce the impact of bias on both the data collected, and the interpretation of the findings [29]. Data analysis will be conducted using NVivo 12 Pro qualitative data analysis software.

Patient and public involvement

A major component of the eP Opt project is the involvement of patient and public representatives across the four project phases. Specifically, two patient and public (PPI) representatives are involved as team members, who attend research meetings and public events to provide feedback and suggestions on the work within each phase from a patient's perspective. They have also been extensively involved with assisting with the design of an upcoming PPI roundtable event for phase 3 of the eP Opt project, where current progress of the present study will be shared with a group of

For peer review only - http://bmjopen.bmj.com/site/about/guidelines.xhtml

invited patients, and their feedback gathered to be used as the study progresses. These roundtable events and PPI consultations have helped in the formulation of research questions and will feed into analysis of the data by highlighting current gaps in practice from a patient perspective.

Study timeline

Benchmark site recruitment started in autumn 2019. All six identified sites were initially contacted in by the end of 2019 and access has been granted to begin recruitment and conduct interviews in four of the six sites. At present, we plan to continue recruitment for sites and individuals iteratively until at least 10 participants have been recruited from each site. Data collection commenced in March 2020, and is anticipated to be completed by early 2021. We have experienced delays in recruitment as access to hospital staff and availability was affected by COVID-19. We aim to have data analysis completed by February 2021, with dissemination of findings anticipated in early 2021.

Discussion

Optimisation of ePrescribing systems can be seen as an ongoing process of identifying gaps in functionality of an already implemented system and negotiating changes, which would address these gaps, and is a complex, multifaceted process. The qualitative study described in this protocol, sits within a multi-phase research project that aims to comprehensively investigate approaches to take when optimising ePrescribing systems, and to highlight important lessons to be learned as ePrescribing continues to be implemented at scale within the NHS [2]. This study will gather the insight of healthcare professionals at leading benchmark sites both in the UK and internationally. All phases of the wider eP Opt project investigate the journey from implementation of an EHR with associated ePrescribing functionality to these systems performing as an optimal resource for professionals and patients. This addresses the important question of unintended consequences of ePrescribing systems, and attempts to ensure that the safety of prescribing processes is not adversely affected as improvements are made.

<u>Author's contributions</u>

AS conceptualised the project and designed the study. CH recruited study sites; CH and SM recruited individual participants within study sites and conducted data collection. CH prepared the first draft of the manuscript. SM and CH prepared the second draft of the manuscript. All authors contributed to the final draft of the manuscript and approved it for submission.

<u>Acknowledgements</u>

The Authors thank all study sites and participants who are currently participating in the project, in addition to the PPI representatives Jillian Beggs and Antony Chuter. We also thank colleagues at the Usher Institute who have contributed to the wider phases of the eP Opt project (Serena Tricarico, Toni Wigglesworth Jac Williams, Mike Holder, Uditha Perera and Matt Bouamrane).

<u>Funding statement</u>

This study/project is funded by the National Institute for Health Research (NIHR) [Optimising ePrescribing in Hospitals (PR-ST-01-10001)/Policy Research Programme]. The views expressed are

For peer review only

those of the author(s) and not necessarily those of the NIHR or the Department of Health and Social Care.

Data statement

Data are available upon reasonable request

Competing interests

The authors declare no conflicts of interest

Figure 1. The four phases of the eP Opt project and interactions between phases, demonstrating how Phase 2 is situated within the wider project.

Ethics and dissemination

This study was granted ethical approval by the Usher Research Ethics Group (University of Edinburgh) on 21/01/2020 (ref.1906) and the relevant NHS research and development approvals were acquired for UK-based sites on 23/01/2020 (ref.19/HRA/7015). As this research is exclusively being conducted with healthcare professionals, there is no involvement of vulnerable groups, and therefore there is minimal ethical concern. Despite this, there are a number of data protection and privacy considerations that should be acknowledged. Firstly, due to the ongoing COVID-19 pandemic, this research project is being conducted remotely using teleconferencing software. This presents a number of potential data protection issues, such as the potential for unauthorised access to scheduled virtual meetings. This issue is being mitigated by using only University-approved accounts to set up interviews, using meeting passwords, and ensuring interviews are undertaken with both the interviewee and interviewer in a private room. Another ethical consideration is the need to protect anonymity of research participants. As we are focusing this research on well-known, world-leading institutions in health informatics, extra care should be taken when presenting research findings to not compromise the anonymity of the respondents. Therefore, specific job titles will not be used in conjunction with individual quotes, and any quotes which could be used to potentially identify someone will not be used. All audio files, transcripts and consent forms will be kept in a secure, password-protected folder within the University of Edinburgh's Datastore. Consent forms will be kept in separate folders from the rest of the research data.

References

1. Catwell L, Sheikh A. Evaluating eHealth interventions: the need for continuous systemic evaluation. *PLoS Med* 2009;6(8):e1000126.
2. NHS England. The NHS long term plan, 2019.
3. Healthcare Safety Investigation Branch. Electronic Prescribing and Medicines Administration Systems and Safe Discharge, 2019:11.
4. Bell H, Garfield S, Khosla S, et al. Mixed methods study of medication-related decision support alerts experienced during electronic prescribing for inpatients at an English hospital. *European Journal of Hospital Pharmacy* 2019;26(6):318-22.

For peer review only

5. OECD European Observatory on Health Systems Policies. Denmark: Country Health Profile 20192019.
6. Garfield S, Jani Y, Jheeta S, et al. Impact of electronic prescribing on patient safety in hospitals: implications for the UK. *Evaluation* 2020;15:13.
7. Cresswell KM, Mozaffar H, Lee L, et al. Safety risks associated with the lack of integration and interfacing of hospital health information technologies: a qualitative study of hospital electronic prescribing systems in England. *BMJ Quality & Safety* 2017;26(7):530-41.
8. Cornford T, Dean B, Savage I, et al. Electronic prescribing in hospitals-challenges and lessons learned. 2009
9. Robertson A, Bates DW, Sheikh A. The rise and fall of England's National Programme for IT: SAGE Publications Sage UK: London, England, 2011.
10. Wachter R. Making IT work: harnessing the power of health information technology to improve care in England. *London, UK: Department of Health* 2016
11. Cresswell KM, Bates DW, Sheikh A. Ten key considerations for the successful optimization of large-scale health information technology. *Journal of the American Medical Informatics Association* 2017;24(1):182-87.
12. Wiegel V, King A, Mozaffar H, et al. A systematic analysis of the optimization of computerized physician order entry and clinical decision support systems: A qualitative study in English hospitals. *Health Informatics Journal* 2019:1460458219868650.
13. Simon SR, Keohane CA, Amato M, et al. Lessons learned from implementation of computerized provider order entry in 5 community hospitals: a qualitative study. *BMC medical informatics and decision making* 2013;13(1):67.
14. Cresswell K, Coleman J, Slee A, et al. Investigating and learning lessons from early experiences of implementing ePrescribing systems into NHS hospitals: a questionnaire study. *PLoS One* 2013;8(1)
15. National Information Board. Personalised health and care 2020: using data and technology to transform outcomes for patients and citizens: a framework for action: HM Government London, 2014.
16. Cresswell K, Sheikh A, Krasuska M, et al. Reconceptualising the digital maturity of health systems. *The Lancet Digital Health* 2019;1(5):e200-e01.
17. NHS England. NHS digital academy., 2018.
18. Williams R, Cresswell K. Independent Evaluation of the Global Digital Exemplar (GDE) Programme. 2020
19. Castle-Clarke S, Hutchings R. Achieving a digital NHS.
20. Tong A, Sainsbury P, Craig J. Consolidated criteria for reporting qualitative research (COREQ): a 32-item checklist for interviews and focus groups. *International journal for quality in health care* 2007;19(6):349-57.
21. Arksey H, O'Malley L. Scoping studies: towards a methodological framework. *International journal of social research methodology* 2005;8(1):19-32.
22. Levac D, Colquhoun H, O'Brien KK. Scoping studies: advancing the methodology. *Implementation science* 2010;5(1):69.
23. Munn Z, Peters MD, Stern C, et al. Systematic review or scoping review? Guidance for authors when choosing between a systematic or scoping review approach. *BMC medical research methodology* 2018;18(1):143.
24. Williams J, Bates DW, Sheikh A. Optimising electronic prescribing in hospitals: a scoping review protocol. *BMJ Health & Care Informatics* 2020;27(1)
25. Flick U. Triangulation in qualitative research. *A companion to qualitative research* 2004;3:178-83.
26. Silverman D. Qualitative research: sage 2016.
27. Thomas J, Harden A. Methods for the thematic synthesis of qualitative research in systematic reviews. *BMC Medical Research Methodology* 2008;8(1):45. doi: 10.1186/1471-2288-8-45

For peer review only

28. O'Connor C, Joffe H. Intercoder Reliability in Qualitative Research: Debates and Practical Guidelines. *International Journal of Qualitative Methods* 2020;19:1609406919899220. doi: 10.1177/1609406919899220
29. Berger R. Now I see it, now I don't: Researcher's position and reflexivity in qualitative research. *Qualitative research* 2015;15(2):219-34.

For peer review only

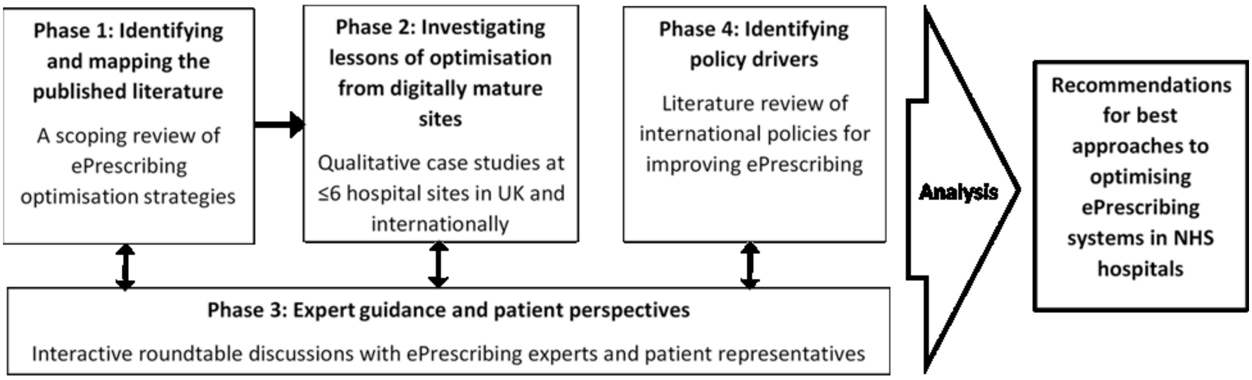

For peer review only

Consolidated criteria for reporting qualitative studies (COREQ): 32-item checklist

Please indicate in which section each item has been reported in your manuscript. If you do not feel an item applies to your manuscript, please enter N/A.

For further information about the COREQ guidelines, please see Tong *et al.*, 2017: https://doi.org/10.1093/intqhc/mzm042

No.	Item	Description	Section #
**Domain 1: Research team and reflexivity**			
Personal characteristics			
1.	Interviewer/facilitator	Which author/s conducted the interview or focus group?	
2.	Credentials	What were the researcher's credentials? *E.g. PhD, MD*	
3.	Occupation	What was their occupation at the time of the study?	
4.	Gender	Was the researcher male or female?	
5.	Experience and training	What experience or training did the researcher have?	
Relationship with participants			
6.	Relationship established	Was a relationship established prior to study commencement?	
7.	Participant knowledge of the interviewer	What did the participants know about the researcher? *E.g. Personal goals, reasons for doing the research*	
8.	Interviewer characteristics	What characteristics were reported about the interviewer/facilitator? *E.g. Bias, assumptions, reasons and interests in the research topic*	
**Domain 2: Study design**			
Theoretical framework			
9.	Methodological orientation and theory	What methodological orientation was stated to underpin the study? *E.g. grounded theory, discourse analysis, ethnography, phenomenology, content analysis*	
Participant selection			
10.	Sampling	How were participants selected? *E.g. purposive, convenience, consecutive, snowball*	
11.	Method of approach	How were participants approached? *E.g. face-to-face, telephone, mail, email*	
12.	Sample size	How many participants were in the study?	
13.	Non-participation	How many people refused to participate or dropped out? What were the reasons for this?	
Setting			
14.	Setting of data collection	Where was the data collected? *E.g. home, clinic, workplace*	
15.	Presence of non-participants	Was anyone else present besides the participants and researchers?	

For peer review only

16.	Description of sample	What are the important characteristics of the sample? *E.g. demographic data, date*	
Data collection			
17.	Interview guide	Were questions, prompts, guides provided by the authors? Was it pilot tested?	
18.	Repeat interviews	Were repeat interviews carried out? If yes, how many?	
19.	Audio/visual recording	Did the research use audio or visual recording to collect the data?	
20.	Field notes	Were field notes made during and/or after the interview or focus group?	
21.	Duration	What was the duration of the interviews or focus group?	
22.	Data saturation	Was data saturation discussed?	
23.	Transcripts returned	Were transcripts returned to participants for comment and/or correction?	
**Domain 3: analysis and findings**			
Data analysis			
24.	Number of data coders	How many data coders coded the data?	
25.	Description of the coding tree	Did authors provide a description of the coding tree?	
26.	Derivation of themes	Were themes identified in advance or derived from the data?	
27.	Software	What software, if applicable, was used to manage the data?	
28.	Participant checking	Did participants provide feedback on the findings?	
Reporting			
29.	Quotations presented	Were participant quotations presented to illustrate the themes / findings? Was each quotation identified? *E.g. Participant number*	
30.	Data and findings consistent	Was there consistency between the data presented and the findings?	
31.	Clarity of major themes	Were major themes clearly presented in the findings?	
32.	Clarity of minor themes	Is there a description of diverse cases or discussion of minor themes?	

When submitting your manuscript via the online submission form, please upload the completed checklist as a Figure/supplementary file.

If you would like this checklist to be included alongside your article, we ask that you upload the completed checklist to an online repository and include the guideline type, name of the repository, DOI and license in the *Data availability* section of your manuscript.

Developed from: Allison Tong, Peter Sainsbury, Jonathan Craig, Consolidated criteria for reporting qualitative research (COREQ): a 32-item checklist for interviews and focus groups, International Journal for Quality in Health Care, Volume 19, Issue 6, December 2007, Pages 349–357, https://doi.org/10.1093/intqhc/mzm042

For peer review only
