# Supplementary material for: Protocol for a qualitative study to identify strategies to optimise hospital ePrescribing systems
Source: BMJ Open. 2021 Jan 13;11(1):e044622. doi: 10.1136/bmjopen-2020-044622 (PMC7812111; doi:10.1136/bmjopen-2020-044622)
Supplement: Reviewer comments [file bmjopen-2020-044622.reviewer_comments.pdf]

PEER REVIEW HISTORY

BMJ Open publishes all reviews undertaken for accepted manuscripts. Reviewers are asked to complete a checklist review form (**http://bmjopen.bmj.com/site/about/resources/checklist.pdf**) and are provided with free text boxes to elaborate on their assessment. These free text comments are reproduced below.

ARTICLE DETAILS

**TITLE (PROVISIONAL)**	Protocol for a qualitative study to identify strategies to optimise hospital ePrescribing systems
**AUTHORS**	Heeney, Catherine; Malden, Stephen; Sheikh, Aziz

This article was not externally reviewed at BMJ Open. Protocols that have been independently assessed prior to submission to BMJ Open are usually fast-tracked to publication on the grounds that further substantial changes will not be possible. This independent assessment will usually be external, independent review for both a substantial grant award from a non-commercial or government funder and ethics approval.
